# Patient Portal Barriers and Group Differences: Cross-Sectional National Survey Study

**DOI:** 10.2196/18870

**Published:** 2020-09-17

**Authors:** Kea Turner, Alecia Clary, Young-Rock Hong, Amir Alishahi Tabriz, Christopher M Shea

**Affiliations:** 1 Department of Health Outcomes and Behavior Moffitt Cancer Center Tampa, FL United States; 2 Center for Healthcare Transformation Avalere Health Washington, DC United States; 3 Department of Health Services Research, Management, and Policy College of Public Health and Health Professions University of Florida Gainesville, FL United States; 4 Division of Pharmaceutical Outcomes and Policy Eshelman School of Pharmacy University of North Carolina at Chapel Hill Chapel Hill, NC United States; 5 Department of Health Policy and Management Gillings School of Global Public Health University of North Carolina at Chapel Hill Chapel Hill, NC United States

**Keywords:** patient portal, personal health record, electronic health record, implementation

## Abstract

**Background:**

Past studies examining barriers to patient portal adoption have been conducted with a small number of patients and health care settings, limiting generalizability.

**Objective:**

This study had the following two objectives: (1) to assess the prevalence of barriers to patient portal adoption among nonadopters and (2) to examine the association between nonadopter characteristics and reported barriers in a nationally representative sample.

**Methods:**

Data from this study were obtained from the 2019 Health Information National Trends Survey. We calculated descriptive statistics to determine the most prevalent barriers and conducted multiple variable logistic regression analysis to examine which characteristics were associated with the reported barriers.

**Results:**

The sample included 4815 individuals. Among these, 2828 individuals (58.73%) had not adopted a patient portal. Among the nonadopters (n=2828), the most prevalent barriers were patient preference for in-person communication (1810/2828, 64.00%), no perceived need for the patient portal (1385/2828, 48.97%), and lack of comfort and experience with computers (735/2828, 25.99%). Less commonly, individuals reported having no patient portal (650/2828, 22.98%), no internet access (650/2828, 22.98%), privacy concerns (594/2828, 21.00%), difficulty logging on (537/2828, 18.99%), and multiple patient portals (255/2828, 9.02%) as barriers. Men had significantly lower odds of indicating a preference for speaking directly to a provider compared with women (odds ratio [OR] 0.75, 95% CI 0.60-0.94; *P*=.01). Older age (OR 1.01, 95% CI 1.00-1.02; *P*<.001), having a chronic condition (OR 1.83, 95% CI 1.44-2.33; *P*<.001), and having an income lower than US $20,000 (OR 1.61, 95% CI 1.11-2.34; *P*=.01) were positively associated with indicating a preference for speaking directly to a provider. Hispanic individuals had significantly higher odds of indicating that they had no need for a patient portal (OR 1.59, 95% CI 1.24-2.05; *P*<.001) compared with non-Hispanic individuals. Older individuals (OR 1.05, 95% CI 1.04-1.06; *P*<.001), individuals with less than a high school diploma (OR 3.15, 95% CI 1.79-5.53; *P*<.001), and individuals with a household income of less than US $20,000 (OR 2.78, 95% CI 1.88-4.11; *P*<.001) had significantly higher odds of indicating that they were uncomfortable with a computer.

**Conclusions:**

The most common barriers to patient portal adoption are preference for in-person communication, not having a need for the patient portal, and feeling uncomfortable with computers, which are barriers that are modifiable and can be intervened upon. Patient characteristics can help predict which patients are most likely to experience certain barriers to patient portal adoption. Further research is needed to tailor implementation approaches based on patients’ needs and preferences.

## Introduction

Patient portals have demonstrated promise in improving patient engagement and outcomes [1-7] but remain underutilized [8-13]. Patient portals (web applications tethered to the electronic health record [EHR]) offer patients numerous ways to better engage in their own care, such as viewing and downloading health information and securely messaging their health care providers [14-16]. To encourage patient portal use among patients and providers, the US government has taken steps to promote patient portal adoption [17,18]. Stage 2 of the Meaningful Use EHR Incentive Program, which is now part of the Merit-based Incentive Payment Program, requires eligible providers to ensure that a certain percentage of patients are downloading and viewing health information and securely messaging their care team [17,19]. Eligible hospitals are also required to ensure that patients download and view health information under the Promoting Interoperability for Hospitals Program. Additionally, the 21st Century Cures Act encourages providers to offer access to patient portals through digital health applications in order to enhance a patient’s ability to maintain a longitudinal health record (ie, integrate portal data from multiple providers) and to share the record with other health care providers [18]. Despite these policy initiatives, patient portal adoption has been slow.

Studies have consistently shown that patient portal usage has increased over time but remains low overall [10,20,21]. A recent nationally representative study (weighted n=254,183 individuals) found a significant increase in the adoption of patient portals in the United States, from 12.5% in 2011 to 25.0% in 2017 (*P*<.001) [10]. However, an overall adoption rate of 25% is modest and means that many patients are still not using patient portals. To increase adoption, some health care systems have started offering access to patient portals through smartphone apps [22]. Smartphone app access allows patients to integrate data from multiple patient portals and may increase access for patients who do not have access to a home computer. However, a recent study examining patient portal access through a smartphone app found that the rate of new users did not significantly change over time (*P*=.18) and that the proportion of patient portal adopters who logged into the smartphone app was low (population mean [] 0.7%, SD 0.2%-2.1%) [23]. This finding suggests that there are other barriers that are affecting patient portal adoption and that additional implementation strategies, beyond accessibility through smartphone apps, may be needed to enhance adoption.

Several studies have identified patient- and provider-level barriers that are associated with low adoption of patient portals. Studies have consistently shown that lower socioeconomic status, older age, rural residence, male gender, black race, Hispanic ethnicity, and public or no insurance are associated with lower adoption of patient portals [8,24-30]. On the other hand, patients with a usual source of care, those having better patient-provider communication, and those with multiple chronic conditions are more likely to adopt patient portals [29,31,32]. Studies have also enumerated many barriers to adoption, such as computer literacy, lack of internet access, privacy concerns, difficulty logging in, and presence of different portals for different providers [33-43]. Many of these studies, however, involved small samples, limiting the ability to discern which barriers are most prominent and which patient subgroups are most likely to experience a specific barrier.

To address this gap, this study had the following two objectives: (1) to assess the prevalence of barriers to patient portal adoption among nonadopters and (2) to examine the association between nonadopter characteristics and reported barriers in a nationally representative sample. By clarifying which barriers are most common and which patient subgroups are most affected by these barriers, future studies can develop targeted implementation approaches to advance patient portal adoption.

## Methods

### Study Design

This was a cross-sectional observational study conducted in 2019. The unit of analysis was the individual.

### Data

Data for this study were obtained from the Health Information National Trends Survey (HINTS), which is administered by the National Cancer Institute (NCI). The HINTS collects data on individuals’ use of and access to health-related information, and health-related knowledge, awareness, and behaviors. The sampling frame included all civilian noninstitutionalized adults (aged over 18 years) living in the United States, and it was considered a nationally representative sample. The sampling strategy was two-staged. First, a stratified sample was selected based on a file of residential addresses maintained by the NCI, and then, one adult within each selected household was sampled. The HINTS 5, Cycle 3 survey, which was used for this study, was administered from January through April 2019 via a paper-based survey and an experimental web survey. The overall response rate was 30.3%.

### Study Population

The HINTS 5, Cycle 3 sample included 5438 individuals. We removed individuals with missing data for key variables (eg, complete case analysis) since the rate of missingness was less than 7% for study variables. We examined whether individuals with missing data were more likely to report nonadoption of patient portals and did not find a relationship; therefore, we concluded that the data were missing at random. We excluded individuals who reported not visiting a health care provider in the past 12 months (n=542). After removing individuals with missing data and individuals who had not visited a provider in the past 12 months, the analytic sample included 4815 individuals (weighted n=227,463,350).

### Measures

#### Adoption of Patient Portals

We divided the sample based on adoption and nonadoption of patient portals according to a survey question (“How many times did you access your online medical record in the last 12 months?”). Individuals selecting zero times were categorized as “nonadopters” and individuals selecting one or more times were categorized as “adopters.”

#### Barriers to Patient Portal Adoption

The survey asked a series of yes/no questions eliciting reasons why patients have not adopted patient portals, including patient preference to speak to a health care provider directly, lack of internet access, concerns about privacy, lack of patient portals, trouble remembering passwords, lack of experience with computers, and having more than one patient portal. Participants were allowed to select more than one barrier. Each of these barriers was categorized as a binary variable.

#### Individual Characteristics

The survey captured several measures of patient characteristics. We included characteristics associated with patient portal adoption that have been reported in previous studies [8,24-30], including binary measures of gender, black race, Hispanic ethnicity, marital status, insurance status, rural residence, presence of a chronic condition (eg, diabetes, hypertension, and heart disease), and having a regular provider. We included age as a continuously measured variable. We also included three categorical variables, including income (eg, less than US $20,000, US $20,000-$34,999, US $35,000-$49,999, US $50,000-$74,999, and US $75,000 or more), education (eg, less than high school diploma, high school diploma, college degree, and postgraduate degree), and satisfaction with care (eg, excellent, very good, good, fair, and poor). Each of these variables has been shown to be a predictor of patient portal adoption in previous research [8,24-30].

### Analytic Approach

First, we described the characteristics of the study population, conducting bivariate analyses to compare the characteristics of the adopters and nonadopters of patient portals. Second, we adopted a series of multiple logistic regression models to examine which characteristics were associated with barriers that were experienced by at least 10% of participants. We chose 10% as a cutoff to ensure that we had an adequate sample size for each logistic regression. We present the results for the three most common barriers in the Results section and provide the results for the less common barriers in Multimedia Appendix 1 and Multimedia Appendix 2. The statistical analyses were conducted using Stata (version 16; StataCorp). We used jackknife replicate weights to account for the complex survey design (ie, stratified cluster sample) in the variance calculations. We also applied sampling weights to develop nationally representative estimates. We adhered to the guidelines for weighting and variance estimation from the NCI [44]. To ensure adequate reporting of our study, we followed the Strengthening the Reporting of Observational Studies in Epidemiology (STROBE) statement [45]. This study was exempted by the Advarra Institutional Review Board owing to the use of publicly available data.

## Results

### Sample Characteristics

The sample included 4815 individuals (weighted n=227,463,350). The majority of participants were female (2746/4815, 57.03%), white (3755/4815, 77.99%), and non-Hispanic (4024/4815, 83.57%) (Table 1). The mean age was 56.3 years (SD 16.7 years). Less than half of the participants had a college degree (1307/4815, 27.14%) or postgraduate degree (963/4815, 20.00%) and a household income of US $75,000 or more per year (1719/4815, 35.70%).

There were more individuals who had not adopted a patient portal (ie, nonadopters; 2828/4815, 58.73%) than individuals who had adopted a patient portal (ie, adopters; 1987/4815, 41.27%) (Table 1). Nonadopters were significantly more likely to be male (*P*<.001), be of black race (*P*=.001), have Hispanic ethnicity (*P*=.02), have lower education attainment (*P*<.001), and have lower income (*P*<.001). Nonadopters were also significantly more likely to live in a rural area (*P*=.002), be unmarried (*P*=.001), be uninsured (*P*=.001), not have a usual source of care (*P*<.001), and rate their quality of care lower (*P*<.001) compared with adopters. Age and having a chronic condition did not significantly impact the decision to adopt a patient portal.

**Table 1:** Sample characteristics.

Characteristic		Total population (N=4815; weighted: 227,463,350)	Adopters (N=1987; weighted: 90,644,145)	Nonadopters (N=2828; weighted: 136,800,000)	*P* value
**Gender, n (%)**					<.001
	Male	2069 (42.97%)	781 (39.31%)	1288 (45.54%)	
	Female	2746 (57.03%)	1206 (60.69%)	1540 (54.46%)	
**Race, n (%)**					.001
	Black	772 (16.03%)	255 (12.83%)	517 (18.28%)	
	White	3755 (77.99%)	1649 (82.99%)	2106 (74.47%)	
**Ethnicity, n (%)**					.02
	Hispanic	791 (16.43%)	265 (13.34%)	526 (18.60%)	
	Non-Hispanic	4024 (83.57%)	1722 (86.66%)	2302 (81.40%)	
Age, µ^a^ (SD)		56.3 (16.79)	54.1 (16.01)	57.8 (17.03)	.08
**Education, n (%)**					<.001
	Less than HS^b^	280 (5.82%)	28 (1.41%)	252 (8.91%)	
	HS diploma	2265 (47.04%)	739 (37.19%)	1526 (53.96%)	
	College degree	1307 (27.14%)	651 (32.76%)	656 (23.20%)	
	Postgraduate degree	963 (20.00%)	569 (28.64%)	394 (13.93%)	
**Income, n (%)**					<.001
	Less than US $20,000	762 (15.83%)	154 (7.75%)	608 (21.50%)	
	US $20,000 to $34,999	547 (11.36%)	164 (8.25%)	383 (13.54%)	
	US $35,000 to $49,999	575 (11.94%)	233 (11.73%)	342 (12.09%)	
	US $50,000 to $74,999	786 (16.32%)	363 (18.27%)	423 (14.96%)	
	US $75,000 or more	1719 (35.70%)	929 (46.75%)	790 (27.93%)	
**Rural, n (%)**					.002
	Yes	521 (10.82%)	169 (8.51%)	352 (12.48%)	
	No	4294 (89.18%)	1818 (91.49%)	2476 (87.55%)	
**Marital status, n (%)**					.001
	Married	2656 (55.16%)	1258 (63.31%)	1398 (49.43%)	
	Unmarried	2159 (44.84%)	729 (36.69%)	1430 (50.57%)	
**Chronic condition, n (%)**					.06
	Yes	2633 (54.68%)	1051 (52.89%)	1582 (55.94%)	
	No	2182 (45.32%)	936 (47.11%)	1246 (44.06%)	
**Insurance status, n (%)**					.001
	Insured	4559 (94.68%)	1941 (97.68%)	2618 (92.57%)	
	Uninsured	256 (5.32%)	46 (2.32%)	210 (7.42%)	
**Regular provider, n (%)**					<.001
	Yes	3392 (70.45%)	1614 (81.23%)	1778 (62.87%)	
	No	1423 (29.55%)	373 (18.77)	1050 (37.13%)	
**Quality of care, n (%)**					<.001
	Excellent	1829 (37.99%)	764 (38.45%)	1065 (37.66%)	
	Very good	1806 (37.51%)	797 (40.11%)	1009 (35.68%)	
	Good	862 (17.90%)	344 (17.31%)	518 (18.32%)	
	Fair	197 (4.91%)	70 (3.52%)	127 (4.49%)	
	Poor	120 (2.49%)	12 (0.60%)	108 (3.82%)	

^a^µ: population mean.

^b^HS: high school

### Prevalence of Barriers to Patient Portal Adoption Among Nonadopters

Among nonadopters (n=2828), the most prevalent barrier to patient portal adoption was patient preference for in-person communication (1810/2828, 64.00%) (Table 2). The second most common barrier was no perceived need for the patient portal (1385/2828, 48.97%). The third most common barrier was lack of comfort and experience with computers (735/2828, 25.99%). Less commonly, individuals reported having no patient portal (650/2828, 22.98%), no internet access (650/2828, 22.98%), privacy concerns (594/2828, 21.00%), difficulty logging on (537/2828, 18.99%), and multiple patient portals (255/2828, 9.02%) (Multimedia Appendix 1 and Multimedia Appendix 2).

### Nonadopter Characteristics and Barriers to Patient Portal Adoption

For the first barrier, men had significantly lower odds of indicating a preference for speaking directly to a provider compared with women (odds ratio [OR] 0.75, 95% CI 0.60-0.94; *P*=.01) (Table 2). Conversely, older age (OR 1.01, 95% CI 1.00-1.02; *P*<.001), having a chronic condition (OR 1.83, 95% CI 1.44-2.33; *P*<.001), having a regular provider (OR 1.45, 95% CI 1.14-1.84; *P*=.003), and having an income lower than US $20,000 (OR 1.61, 95% CI 1.11-2.34; *P*=.01) were positively associated with indicating a preference for speaking directly to a provider. In terms of education, individuals with less than a high school education (OR 2.03, 95% CI 1.17-3.50; *P*=.011), a high school diploma (OR 2.16, 95% CI 1.57-2.97; *P*<.001), and a college degree (OR 1.40, 95% CI 1.01-1.94; *P*=.04) had significantly higher odds of preferring to speak directly to a provider compared with individuals having a postgraduate degree. Individuals who rated their quality of care as “very good” (OR 0.58, 95% CI 0.36-0.95; *P*=.03) or “good” (OR 0.64, 95% CI 0.47-0.87; *P*=.004) had significantly lower odds of preferring to speak directly to a provider compared with individuals who rated their quality of care as “poor.”

For the second barrier, Hispanic individuals had significantly higher odds of indicating that they had no need for a patient portal (OR 1.59, 95% CI 1.24-2.05; *P*<.001) compared with non-Hispanic individuals (Table 2). In contrast, older individuals (OR 0.99, 95% CI 0.99-1.00; *P*=.04), individuals who rated their quality of care as “excellent” (OR 0.33, 95% CI 0.12-0.88; *P*=.03), and individuals with a household income of US $20,000 to $34,999 (OR 0.61, 95% CI 0.44-0.85; *P*=.003) or less than US $20,000 (OR 0.68, 95% CI 0.50-0.93; *P*=.02) had significantly lower odds of indicating that they had no need for a patient portal.

For the third barrier, older individuals (OR 1.05, 95% CI 1.04-1.06; *P*<.001), individuals with a chronic condition (OR 1.42, 95% CI 1.08-1.86; *P*=.01), and individuals who rated their quality of care as “good” (OR 1.55, 95% CI 1.11-2.15; *P*=.009) had significantly higher odds of indicating that they were uncomfortable with a computer (Table 2). Individuals with less than a high school diploma (OR 3.15, 95% CI 1.79-5.53; *P*<.001) and a high school diploma (OR 2.79, 95% CI 1.80-4.33; *P*<.001) had significantly higher odds of indicating that they were uncomfortable with a computer. Individuals with a household income of less than US $20,000 (OR 2.78, 95% CI 1.88-4.11; *P*<.001), US $20,000 to $34,999 (OR 2.17, 95% CI 1.45-3.27; *P*<.001), US $35,000 to $49,999 (OR 1.94, 95% CI 1.30-2.90; *P*=.001), and US $50,000 to $74,999 (OR 1.57, 95% CI 1.30-2.90; *P*=.001) had significantly higher odds of indicating that they were uncomfortable with a computer compared with individuals having a household income of US $75,000 or more. Black individuals (OR 0.93, 95% CI 0.89-0.98; *P*=.007) were less likely to indicate that they were uncomfortable with a computer compared with white individuals.

**Table 2:** Nonadopter characteristics and the three most common barriers to patient portal adoption.

Characteristic		Model 1: Speaking directly to a provider (N=1926; weighted: 88,630,105)			Model 2: No need for a patient portal (N=1893; weighted: 87,531,372)			Model 3: Uncomfortable with a computer (N=1893; weighted: 87,531,372)		
		OR^a^	95% CI	*P* value	OR	95% CI	*P* value	OR	95% CI	*P* value
**Gender**										
	Male	0.75	0.60-0.94	.01	1.03	0.85-1.25	.75	1.18	0.93-1.49	.18
	Female (ref^b^)	N/A^c^	N/A	N/A	N/A	N/A	N/A	N/A	N/A	N/A
**Race**										
	Black	1.01	0.96-1.06	.73	1.05	1.00-1.10	.05	0.93	0.89-0.98	.007
	White (ref)	N/A	N/A	N/A	N/A	N/A	N/A	N/A	N/A	N/A
**Ethnicity**										
	Hispanic	0.90	0.67-1.21	.48	1.59	1.24-2.05	<.001	0.81	0.60-1.10	.19
	Non-Hispanic (ref)	N/A	N/A	N/A	N/A	N/A	N/A	N/A	N/A	N/A
Age		1.01	1.00-1.02	<.001	0.99	0.99-1.00	.04	1.05	1.04-1.06	<.001
**Education**										
	Less than HS^d^	2.03	1.17-3.50	.01	0.68	0.43-1.07	.10	3.15	1.79-5.53	<.001
	HS diploma	2.16	1.57-2.97	<.001	0.98	0.73-1.32	.91	2.79	1.80-4.33	<.001
	College degree	1.40	1.01-1.94	.04	1.1	0.81-1.50	.55	1.39	0.86-2.26	.18
	Postgraduate (ref)	N/A	N/A	N/A	N/A	N/A	N/A	N/A	N/A	N/A
**Income**										
	Less than US $20,000	1.61	1.11-2.34	.01	0.68	0.50-0.93	.02	2.78	1.88-4.11	<.001
	US $20,000 to $34,999	1.32	0.90-1.94	.16	0.61	0.44-0.85	.003	2.17	1.45-3.27	<.001
	US $35,000 to $49,999	0.97	0.68-1.38	.86	0.79	0.58-1.08	.14	1.94	1.30-2.90	< .001
	US $50,000 to $74,999	1.19	0.86-1.64	.29	0.99	0.74-1.32	.92	1.57	1.06-2.31	.02
	US $75,000 or more (ref)	N/A	N/A	N/A	N/A	N/A	N/A	N/A	N/A	N/A
**Rural**										
	Yes	1.05	0.74-1.49	.78	1.06	0.79-1.42	.70	1.02	0.72-1.45	.91
	No (ref)	N/A	N/A	N/A	N/A	N/A	N/A	N/A	N/A	N/A
**Marital status**										
	Married	1.04	0.82-1.32	.74	0.86	0.69-1.06	.16	1.20	0.93-1.56	.16
	Unmarried (ref)	N/A	N/A	N/A	N/A	N/A	N/A	N/A	N/A	N/A
**Chronic condition**										
	Yes	1.83	1.44-2.33	<.001	0.93	0.75-1.16	.53	1.42	1.08-1.86	.01
	No (ref)	N/A	N/A	N/A	N/A	N/A	N/A	N/A	N/A	N/A
**Insurance status**										
	Uninsured	0.88	0.53-1.47	.63	0.85	0.53-1.35	.49	0.60	0.31-1.15	.12
	Insured (ref)	N/A	N/A	N/A	N/A	N/A	N/A	N/A	N/A	N/A
**Regular provider**										
	Yes	1.45	1.14-1.84	.003	1.19	0.96-1.48	.12	.98	0.75-1.30	.92
	No (ref)	N/A	N/A	N/A	N/A	N/A	N/A	N/A	N/A	N/A
**Quality of care**										
	Excellent	0.52	0.19-1.42	.20	0.33	0.12-0.88	.03	1.76	0.62-4.99	.29
	Very good	0.58	0.36-0.95	.03	0.69	0.44-1.08	.10	1.41	0.83-2.39	.21
	Good	0.64	0.47-0.87	.004	0.92	0.71-1.20	.54	1.55	1.11-2.15	.009
	Fair	0.93	0.71-1.22	.60	0.99	0.79-1.24	.94	1.09	0.82-1.46	.54
	Poor (ref)	N/A	N/A	N/A	N/A	N/A	N/A	N/A	N/A	N/A
Constant		0.66	0.32-1.36	.26	0.94	0.50-1.77	.86	0.01	0.00-0.01	<.001

^a^OR: odds ratio.

^b^ref: reference.

^c^N/A: not applicable.

^d^HS: high school.

## Discussion

### Principal Findings

The goal of this study was to assess the prevalence of barriers to patient portal adoption in a nationally representative sample and to understand which patient subgroups are most likely to experience a given barrier. Our study found that the most common barriers to patient portal adoption are an individual’s preference to speak to a provider in person, not having a need for the patient portal, and feeling uncomfortable with computers, which are barriers that are modifiable and can be intervened upon. Less frequently, patients reported concerns with privacy, internet access, difficulty logging on, and having multiple patient portals. Our study identified that patient characteristics can help predict which patients are most likely to experience certain barriers to patient portal adoption. Older adults and women, for example, commonly reported preference for in-person communication as a barrier to patient portals. We discuss implications for policy and practice below.

Consistent with previous studies, our study indicated that patient preference for in-person communication with providers serves as a barrier to patient portal adoption [38,46-49]. This study extends prior studies by demonstrating that this barrier may be more common (1810/2828, 64.00%) than previously thought and that certain patient demographics are associated with preferring in-person communication (eg, women, older patients, and patients with lower income and education). Dissemination strategies, which target information to a specific audience, may be needed to demonstrate that patient portals are meant to complement rather than replace in-person communication with providers [50]. For developing better messaging, however, there is a need for more implementation studies identifying effective practices for using the patient portal as a means to bolster patient-physician communication during visits. There have been successful examples of this, including collecting and displaying patient-reported outcomes through the portal, and using the portal to facilitate advanced care planning discussions [12,38,51-53]. These strategies should be replicated in additional settings.

Almost half of the individuals in our study indicated that not having a need for a patient portal was a barrier to adoption, which has emerged as a barrier in past research [33,34,36,37]. Our study found that Hispanic and younger individuals were more likely to not see a need for the patient portal. A prior study recommended using real-life patient stories that demonstrate how a patient portal can be used to make the case for why a patient portal might be valuable [54]. There also remains a need to do more usability testing with patient portals and apply user-centered design approaches to better understand what features within the patient portal would be valuable to patients [55-57]. Studies have identified a number of usability issues, including not having information presented in multiple languages, lack of educational resources, poor data visualization and lack of contextualization for laboratory values, and lack of personalization [58-60]. In response, some systems have tested creative strategies, such as offering tailored patient education, and have used motivational strategies (eg, social comparisons and gamification) to enhance the relevance of the patient portal and ensure that the portal is meeting user needs [55,61]. Future studies are needed to test strategies that align the patient portal with patients’ information needs. Some systems have also trained providers on the portal and created time within clinic workflow to show patients what the portal is and how to use it, and these strategies may enhance patients’ perceived need for the portal [62,63]. Further testing is needed to see whether this is an effective implementation approach.

Our study also found that lack of comfort with computers (735/2828, 25.99%) was a common barrier to patient portal adoption, a finding similar to that in past studies [33-35,37,64-66]. Consistent with prior work, we found that older individuals and individuals with lower income and education attainment were more likely to report lack of comfort with computers as a barrier to patient portal adoption. Several studies have tested strategies, such as having health care systems offer patient portal demonstrations to patients, as means to increase comfort with computers and ultimately patient portal adoption [2,62,67]. Researchers have also recommended providing additional support to older individuals who may lack comfort with computers, such as printed handouts and an option to call a toll free line for additional technical assistance [67,68]. Past studies have found that in-person training can improve eHealth literacy among older adults [69-71]; however, few studies have been performed in clinical settings or have included clinical outcomes. Thus, additional research is warranted. Past studies have suggested that some older individuals experience additional barriers to technology adoption, such as vision, cognitive, and dexterity deficits [72]. Future studies should test whether modifications to patient portals, such as larger font size, increased contrast between the text and background, and voice-enabled applications, could increase comfort with patient portals among older frail adults. Some health care systems have also allowed patients to designate a caregiver to access the patient portal on their behalf, although uptake has been slow [73-75]. Implementation strategies that incorporate patient caregivers, such as proxy portal access, training for caregivers on the patient portal, and allowing patients to choose which information is shared with the caregiver, could help make the portal more accessible to patients who lack comfort with technology.

### Limitations

This study has a number of limitations. First, the HINTS does not collect any information at the site of care where a patient is seen. It is possible that system- and provider-level factors influence which patients are most likely to experience certain barriers to patient portal adoption. For example, patients who receive care in a Veterans Affairs hospital may be more likely to report a need for the patient portal since the Veterans Affairs has been an early adopter of patient portals [57]. Additionally, there are other barriers that may affect patient portal adoption, such as lack of reimbursement for telemedicine. These factors were not captured in the HINTS. Second, the HINTS only asked about a limited set of barriers, and it is impossible to tell whether there are other barriers that may be more impactful in hindering patient portal adoption (eg, lack of Spanish language options for the patient portal). Further, the HINTS did not use an implementation framework to select questions related to patient portal barriers. It is possible that other important barriers may have been omitted. The survey questions do, however, capture many of the barriers reported in prior portal studies [38,46-49], suggesting that the questions align well with prior research. Third, the HINTS response rate was around 30%, so it is possible that the findings are not representative of the entire sample owing to nonresponse bias. Finally, the HINTS does not include a measure of multimorbidity (eg, the Charlson comorbidity index), which is positively associated with patient portal adoption. To address this limitation, we included a variable that indicated whether a patient had a chronic condition.

### Conclusions

To our knowledge, this is the first study to assess the prevalence of barriers to patient portal adoption in a nationally representative sample and to discern which patient subgroups are most likely to experience certain barriers. Further research is needed to develop and test implementation strategies that target common barriers to patient portal adoption and tailor implementation and dissemination approaches based on patients’ needs and preferences.

## Appendix

Multimedia Appendix 1 Nonadopter characteristics and no patient portal or internet access.

Multimedia Appendix 2 Nonadopter characteristics and privacy concerns or difficulty logging on.

## Abbreviations

EHR: electronic health record
HINTS: Health Information National Trends Survey
NCI: National Cancer Institute
OR: odds ratio
