# Supplementary material for: Patient Portal Barriers and Group Differences: Cross-Sectional National Survey Study
Source: J Med Internet Res. 2020 Sep 17;22(9):e18870. doi: 10.2196/18870 (PMC7530687; doi:10.2196/18870)
Supplement: Multimedia Appendix 1 [file jmir_v22i9e18870_app1.docx]

**Appendix 1. Non-adopter characteristics and no patient portal or internet access**

| **Characteristics** | **Model 4:** | | | **Model 5:** | | |
| --- | --- | --- | --- | --- | --- | --- |
|  | No patient portal | | | No Internet access | | |
|  | N: 2,828 | | | N: 2,828 | | |
|  | Weighted: 136, 800,000 | | | Weighted: 136, 800,000 | | |
|  | OR | 95% CI | *P* | OR | 95% CI | *P* |
| **Gender** |  |  |  |  |  |  |
| Male | 1.13 | 0.91-1.41 | .29 | 1.31 | 1.04-1.64 | .02 |
| Female (*ref*) |  |  |  |  |  |  |
| **Race** |  |  |  |  |  |  |
| Black | 0.91 | 0.68-1.22 | .53 | 1.10 | 0.82-1.46 | .53 |
| White (*ref*) |  |  |  |  |  |  |
| **Ethnicity** |  |  |  |  |  |  |
| Hispanic | 1.14 | 0.85-1.52 | .40 | 1.19 | 0.88-1.60 | .27 |
| Non-Hispanic (*ref)* |  |  |  |  |  |  |
| **Age** | 0.99 | 0.99-1.01 | .83 | 1.01 | 1.00-1.02 | .002 |
| **Education** |  |  |  |  |  |  |
| Less than HS | 0.96 | 0.61-1.50 | .85 | 1.04 | 0.67-1.60 | .86 |
| HS diploma | 1.00 | 0.62-1.63 | .99 | 0.87 | 0.54-1.41 | .57 |
| College degree | 0.94 | 0.55-1.61 | .82 | 0.75 | 0.44-1.30 | .31 |
| Postgraduate (*ref)* |  |  |  |  |  |  |
| **Income** |  |  |  |  |  |  |
| Less than $20,000 | 0.89 | 0.61-1.30 | .52 | 1.11 | 0.78-1.57 | .57 |
| $20,000 to $34,999 | 1.35 | 0.93-1.96 | .12 | 0.81 | 0.55-1.19 | .28 |
| $35,000 to $49,999 | 1.11 | 0.77-1.61 | .58 | 0.69 | 0.47-1.00 | .05 |
| $50,000 to $74,999 | 0.98 | 0.68-1.40 | .89 | 0.69 | 0.48-1.00 | .05 |
| $75,000+ (*ref)* |  |  |  |  |  |  |
| **Rural** |  |  |  |  |  |  |
| Yes | 1.05 | 0.75-1.46 | .79 | 1.16 | 0.83-1.63 | .38 |
| No (*ref)* |  |  |  |  |  |  |
| **Marital status** |  |  |  |  |  |  |
| Married | 0.78 | 0.61-0.99 | .04 | 0.84 | 0.66-1.08 | .17 |
| Unmarried (*ref)* |  |  |  |  |  |  |
| **Chronic condition** |  |  |  |  |  |  |
| Yes | 0.94 | 0.74-1.21 | .64 | 1.24 | 0.96-1.61 | .11 |
| No (*ref)* |  |  |  |  |  |  |
| **Insurance status** |  |  |  |  |  |  |
| Uninsured | 2.09 | 1.28-3.40 | .003 | 1.18 | 0.69-2.03 | .54 |
| Insured (*ref)* |  |  |  |  |  |  |
| **Regular provider** |  |  |  |  |  |  |
| Yes | 0.90 | 0.70-1.16 | .41 | 1.04 | 0.80-1.35 | .78 |
| No (*ref)* |  |  |  |  |  |  |
| **Quality of Care** |  |  |  |  |  |  |
| Excellent | 0.44 | 0.16-1.19 | .11 | 0.79 | 0.28-2.26 | .66 |
| Very good | 0.39 | 0.15-0.97 | .04 | 0.76 | 0.29-2.02 | .58 |
| Good | 0.38 | 0.15-0.93 | .04 | 0.64 | 0.24-1.69 | .37 |
| Fair | 0.32 | 0.13-0.79 | .01 | 0.51 | 0.19-1.35 | .18 |
| Poor (*ref)* |  |  |  |  |  |  |
| **Constant** | 1.11 | 0.38-3.21 | .85 | 0.23 | 0.07-0.70 | .01 |
