# Supplementary material for: Patient Portal Barriers and Group Differences: Cross-Sectional National Survey Study
Source: J Med Internet Res. 2020 Sep 17;22(9):e18870. doi: 10.2196/18870 (PMC7530687; doi:10.2196/18870)
Supplement: Multimedia Appendix 2 [file jmir_v22i9e18870_app2.docx]

**Appendix 2: Non-adopter characteristics and privacy concerns or difficulty logging on**

| **Characteristics** | **Model 6:** | | | **Model 7:** | | |
| --- | --- | --- | --- | --- | --- | --- |
|  | Privacy concerns | | | Difficulty logging on | | |
|  | N: 2,828 | | | N: 2,828 | | |
|  | Weighted: 136, 800,000 | | | Weighted: 136, 800,000 | | |
|  | OR | 95% CI | P | OR | 95% CI | P |
| **Gender** |  |  |  |  |  |  |
| Male | 1.09 | 0.78-1.54 | .61 | 0.92 | 0.59-1.42 | .69 |
| Female (*ref*) |  |  |  |  |  |  |
| **Race** |  |  |  |  |  |  |
| Black | 1.34 | 0.77-2.31 | .30 | 1.32 | 0.61-2.86 | .47 |
| White (*ref*) |  |  |  |  |  |  |
| **Ethnicity** |  |  |  |  |  |  |
| Hispanic | 1.57 | 0.85-2.90 | .14 | 0.96 | 0.55-1.67 | .88 |
| Non-Hispanic (*ref)* |  |  |  |  |  |  |
| **Age** | 1.01 | 1.00-1.02 | .03 | 1.00 | 0.99-1.02 | .72 |
| **Education** |  |  |  |  |  |  |
| Less than HS | 1.22 | 0.50-2.92 | .66 | 0.47 | 0.12-1.78 | .26 |
| HS diploma | 0.70 | 0.29-1.66 | .41 | 0.74 | 0.19-2.95 | .67 |
| College degree | 0.8 | 0.29-2.20 | .66 | 0.87 | 0.21-3.69 | .85 |
| Postgraduate (*ref)* |  |  |  |  |  |  |
| **Income** |  |  |  |  |  |  |
| Less than $20,000 | 0.67 | 0.38-1.19 | .17 | 1.42 | 0.75-2.70 | .28 |
| $20,000 to $34,999 | 0.74 | 0.32-1.68 | .46 | 1.2 | 0.39-3.65 | .75 |
| $35,000 to $49,999 | 0.71 | 0.35-1.45 | .34 | 0.67 | 0.38-1.18 | .16 |
| $50,000 to $74,999 | 0.71 | 0.36-1.41 | .32 | 0.68 | 0.38-1.22 | .19 |
| $75,000+ (*ref)* |  |  |  |  |  |  |
| **Rural** |  |  |  |  |  |  |
| Yes | 0.81 | 0.50-1.33 | .40 | 0.75 | 0.43-1.32 | .32 |
| No (*ref)* |  |  |  |  |  |  |
| **Marital status** |  |  |  |  |  |  |
| Married | 1.1 | 0.67-1.78 | .71 | 1.12 | 0.68-1.85 | .65 |
| Unmarried (*ref)* |  |  |  |  |  |  |
| **Chronic condition** |  |  |  |  |  |  |
| Yes | 1.15 | 0.75-1.77 | .53 | 1.80 | 1.13-2.87 | .01 |
| No (*ref)* |  |  |  |  |  |  |
| **Insurance status** |  |  |  |  |  |  |
| Uninsured | 0.6 | 0.15-2.35 | .46 | 0.52 | 0.18-1.46 | .21 |
| Insured (*ref)* |  |  |  |  |  |  |
| **Regular provider** |  |  |  |  |  |  |
| Yes | 1.10 | 0.79-1.53 | .57 | 1.45 | 0.93-2.27 | .10 |
| No (*ref)* |  |  |  |  |  |  |
| **Quality of Care** |  |  |  |  |  |  |
| Excellent | 0.80 | 0.16-4.11 | .79 | 0.27 | 0.06-1.16 | .08 |
| Very good | 0.46 | 0.09-2.32 | .34 | 0.34 | 0.08-1.48 | .15 |
| Good | 0.51 | 0.12-2.20 | .36 | 0.24 | 0.06-0.92 | .04 |
| Fair | 0.50 | 0.10-2.50 | .39 | 0.20 | 0.05-0.81 | .03 |
| Poor (*ref)* |  |  |  |  |  |  |
| **Constant** | 0.27 | 0.05-1.45 | .13 | 0.87 | 0.10-7.56 | .90 |
